# The Book World of Medicine and Science

**Published:** 1894-01-27

**Authors:** 


### The Book World of Medicine and Science.

### BOOK REVIEWS.

Life and Times of the Right Honourable William Henry Smith, M.P. By Sir Herbert Maxwell, Bart., M.P. Two vols. (William Blackwood and Sons, Edinburgh and London.)

This is as interesting a life as it has been our lot to read for some time, and Sir Herbert Maxwell seems in thorough sympathy with his subject, an essential, but not always usual circumstance in recent biography. The son of a well-to-do newsman in Duke Street, Grosvenor Square, William Henry was early in life made to feel that he must follow in his father's footsteps in spite of his own inclination, which tended rather to a University career and Holy Orders. But his family being Wesleyans did not receive his wishes with much favour, and we shortly after find him entered upon the duties of the book and newspaper business with that ungudging thoroughness and sound common sense which were his marked characteristics through life.

All the modern developments of the business are due to him, and the taking over of the bookstalls at the various railway stations throughout the kingdom was entirely due to young Smith's initiative.

The stalls had previously been a curious mixture of apples, gingerbread, and of the lowest type of literature, and Smith *père* looked upon his son's enterprise with an anything but encouraging eye. However, as young Smith's business dealings had hitherto been a success, he was allowed a free hand, and the undertaking soon proved a financial success.

In 1865 he stood for Westminster, but did not get into Parliament until three years later. He began life as a Moderate Liberal, but, owing to various causes, finally took his stand upon the Conservative side. His view of politics seems to have been always a business-like and common sense one, and he never spoke in the House unless he had something to say, and then said it well, though his speeches always lacked that culture and refinement of expression which is considered the especial outcome of a classical education.

He was a man of varied knowledge and experience, and it could never be said of him that he gave "a verdict based on ignorance" upon any subject.

He was a great lover of flowers, and of lilies of the valley especially; at his pretty place near Henley he grew them all the year round—upon ice in summer and in hot houses in winter. He was a member of Lord Beaconsfield's and of Lord Salisbury's Cabinets, and it was upon the occasion of his visiting Cyprus and some of the Greek islands after the Treaty of Berlin with Colonel Stanley, the Minister for War, that a friend wrote the following epigram:—

"The natives amazed to see such fine stars,

Christened one of them Neptune, and t'other one Mars;

They erected an altar to Stanley forthwith,

And put up a bookstall to W. H. Smith."

Shortly before his death, Mr. Smith was made Lord Warden of the Cinque Ports, and here, during his short tenure of office, he set himself to work with all his old energy and thoroughness. Indeed, the guiding principle of his useful life seems to have been "what was worth doing at all, was worth doing well."

Antiseptics in Midwifery. By Robert Boxall, M.D., M.R.C.P. (London: H. K. Lewis.)

This is an opportune little book. Few people will hesitate to admit the desirability of extending to women in childbirth the benefit of those antiseptic methods which have proved of such signal service in all other surgical procedures, if it can be done. Reference, however, to the various text-books on midwifery gives but uncertain guidance as to how this is to be accomplished, there being but little unanimity among them in regard either to the nature of the antiseptic to be used, or the method of its application. Readers will therefore turn with relief to Dr. Boxall's chapter on the "Technique of Antisepsis," in which the necessary proceedings are described with the dogmatic exactness of a receipt book.

The only point in which the rules there laid down seem somewhat to exceed in stringency those generally accepted as necessary in private practice, is in reference to douching the vagina. Even hospital experience has not been universally in favour of this as a routine proceeding. The washing and disinfection of the hands, both of doctor and nurse; the cleaning and disinfection of the external genitals of the patient, both during and after labour; the use of antiseptic pads for the reception of the discharges; the substitution of pledgets of cotton-wool for sponges; the avoidance of frequent examinations; and the careful disinfection of the instruments, are points on which all authors are agreed, but the direction that "in every case the vagina should be douched once during the first stage of labour, and again once after the labour is completed, with sublimate solution, 1 in 2,000," is one which, while appropriate enough in hospitals where there is an aggregation of cases, will certainly provoke criticism from general practitioners in the country, where patients are sparsely scattered, at great distances from one another, and where of necessity such douches would often have to be administered by more or less ignorant nurses.

Dr. Boxall shows, with great force, the large preventible mortality which is still going on from septic diseases in connection with midwifery, and there can be but little doubt that his recommendations, if skilfully carried out, would at once have the effect of saving hundreds of lives, and an untold amount of misery every year. Whether, so far as prevention goes, a careful use of all the other means suggested, without the routine use of the douche at all, would not be equally successful is another question.

The Student's Handbook of Gynæcology, pp. 178. Illustrated. (Edinburgh: E. and S. Livingstone, 1893.)

This book is written for students, says the preface, and we think it would be well for the students had it never been written. There is nothing new or original in the work, being simply one of those small cram books with as many bald facts as can be stifled into 178 pages of a small book with large print. Surely we should have a far higher aim in an important branch of our profession, which is already much too little generally studied, than to provide such a pamphlet as this which will only be made use of by the idle student for purely examination purposes, and who will forget a month later all that he ever read on the subject. It begins, after the anatomical section, with a description of peritonitis, to which important subject it devotes a whole page and a-half; after reading this one has not the faintest idea of what pelvic peritonitis is, and to heighten the absurdity, it winds up with a few lines on the treatment of the disease which it has professed to describe, and actually advises that "the purulent fluid should be tapped through the posterior fornix." Martin's and Schrœder's operations are constantly referred to but never properly described, so the student is left to draw on his imagination, and finally to talk about them, probably before his examiners, who would quickly detect his ignorance. The author's treatment of gonorrhœa is antiquated, and the few sentences written on the electrical treatment of fibroids shows that he does not understand the subject.

